# Impact of HIV Status on Fruit and Vegetable Consumption Among Older Adults in Tanzania: A Cross-Sectional Secondary Data Analysis

**DOI:** 10.3390/nu18030430

**Published:** 2026-01-28

**Authors:** Mary V. Mosha, Heavenlight A. Paulo, Victoria T. Ayodele, Bahati Wajanga, Mirlene Perry, Charles Muiruri

**Affiliations:** 1 Department of Community Medicine, KCMC University, Moshi P.O. Box 2240, Tanzania; maryanfort@yahoo.com; 2 Department of Biostatistics and Epidemiology, Muhimbili University of Health and Allied Sciences, Dar es Salaam P.O. Box 65001, Tanzania; 3 Department of Biostatistics, School of Public Health, Boston University, Boston, MA 02118, USA; 4 Duke Global Health Institute, Duke University, Durham, NC 27708, USA; victoria.ayodele@duke.edu (V.T.A.); mirlene.perry@duke.edu (M.P.); 5 Department of Internal Medicine, Bugando Medical Centre, Mwanza P.O. Box 1370, Tanzania; wajangabmk@yahoo.com; 6 Department of Medicine, Catholic University of Health and Allied Sciences, Mwanza P.O. Box 1464, Tanzania; 7 Duke University School of Nursing, Duke University, Durham, NC 27708, USA; 8 Department of Population Health Sciences, Duke University School of Medicine, Durham, NC 27710, USA

**Keywords:** fruit and vegetable intake, HIV/AIDS, obesity, non-communicable diseases, nutrition, cardiovascular disease, Tanzania, older adults

## Abstract

**Background/Objectives**: It is well documented that people with human immunodeficiency virus (HIV) have nearly twice the risk of incident acute myocardial infarction compared to the general population. The elevated risk stems from a multi-layered interplay of factors such as persistent immune activation inherent to HIV infection and higher prevalence of traditional risk factors associated with nutritional needs. A large proportion of people living with HIV (PWH) reside in Sub-Saharan African countries such as Tanzania; however, there is a dearth of data on nutrition, particularly fruit and vegetable (F&V) intake, a key factor in the prevention of cardiovascular disease (CVD). This study aimed to contribute to the growing literature on CVD prevention for PWH globally. **Methods**: We conducted secondary analyses of original data collected from a study using the World Health Organization (WHO) STEPS survey among PWH and the general population in Mwanza City between December 2018 and May 2019. Approval for the parent study was obtained from Bugando Medical Center. Multinomial logistic regression analysis examined F&V intake and associated factors between PWH and people living without HIV (PWoH) using sex, employment, and BMI. **Results**: A total of 537 participants (277 PWoH and 260 PWH) were included in the analysis. PWH were more likely to consume fruits ≥ 4 days per week than PWoH (38% vs. 25%, *p* = 0.002), whereas vegetable intake did not differ significantly between groups. Fruit intake was higher in males (OR = 5.63; 95% CI: 2.48–12.79) and employed individuals (OR = 3.85; 95% CI: 1.82–8.14). **Conclusions**: PWH were more likely to consume more fruits than PWoH in this study, a phenomenon that is more novel than previous research. These findings are encouraging to support nutrition-based interventions for PWH who are at a higher risk of CVD.

## 1. Introduction

Non-communicable diseases (NCDs) are rising globally, accounting for two-thirds of deaths. Low- and middle-income countries (LMICs) account for the largest share of these deaths. Among NCD-related deaths, cardiovascular diseases (CVDs) are the leading cause (with 18 million annual deaths), followed by cancer, chronic respiratory diseases, and diabetes, respectively. These NCDs share common risk factors- tobacco use, physical inactivity, alcohol abuse, and an unhealthy diet. Concurrently, LMICs have the highest burden of human immunodeficiency virus (HIV)/acquired immunodeficiency syndrome (AIDS). As advancements in antiretroviral therapy (ART) have significantly extended the life expectancy of people living with HIV (PWH), a notable intersection between CVD and HIV has emerged, shedding light on the elevated risk of CVD events among PWH [1,2,3]. Furthermore, extensive cohort studies have found that PWH have a 50% higher risk of incident myocardial infarction compared to the general population. They often fail to meet treatment goals for CVD risk factors [4,5].

Among modifiable risk factors, a healthy diet plays a pivotal role in promoting health and preventing diseases by bolstering the immune system and reducing the risk of CVDs [6]. A recent study in Tanzania showed the importance of plant-based diets in improving various metabolic markers associated with increased risk of NCDs [7]. Unhealthy diets increase risks of obesity, a multifactorial disease that presents a global public health challenge due to its direct contribution to CVDs and other NCDs [8].

Fruit and vegetable intake is associated with lower risks of NCDs, such as CVD and certain types of cancer, and with lower overall mortality [9,10,11,12]. Studies have reported a significant association between F&V intake and the risk of CVDs. Additionally, certain F&V contain naturally occurring compounds, such as flavonoids, known for their antioxidative and anti-inflammatory properties, which may reduce the risks associated with chronic diseases and help manage the symptoms of CVDs [13,14]. Although the benefits of F&V intake are well documented, there is a dearth of data for vulnerable populations, such as PWH, in LMICs. To add to the body of knowledge in these research areas, we aimed to assess whether there were differences in F&V intake between PWH at higher CVD risk by age and unaffected adults of similar older ages. We also evaluated the association of F&V intake with central obesity in Northwestern Tanzania. These data are crucial in the development and implementation of interventions to reduce CVD risk for PWH.

## 2. Materials and Methods

### 2.1. Parent Study Setting

The original cross-sectional parent study leading to this current secondary analysis was conducted between December 2018 and May 2019, before publication in 2022. These methods for this secondary analysis were published in Correlates of blood pressure awareness, treatment, and control among adults 50 years or older by HIV status in Northwestern Tanzania (2022), serving as the foundation for this study that included protocols and data collection details [15]. PWH were recruited from Bugando Medical Center (BMC) HIV Care and Treatment Center (CTC) as a referral hospital serving at least 4000 PWH through check-ups and pharmaceutical refills. Individuals without HIV in Mwanza were recruited in neighborhoods in Nyamagana and Ilemela districts. Recruitment of PWH and PWoH was not always done in parallel from December to May.

### 2.2. Participants

Eligible participants in the parent study prior to this secondary analysis were PWH and those who were not with HIV above 50 years of age, as described in the parent study [15]. The parent study received ethical approval from the Catholic University of Health and Allied Sciences (CREC/214/2017) on 25 July 2017 [15].

### 2.3. Procedure

PWH were identified through appointment registers at the BMC CTC while being informed and providing consent to the parent study. PWH were later referred to a private research office for confirming enrollment. The data was collected in five wards, with meetings preemptively scheduled to inform all participants to gather in on location and receive HIV and NCD counseling prior to official enrollment and consent. Study objectives were in Swahili.

After obtaining informed consent, an adapted version of the World Health Organization (WHO) STEPS Instrument for Non-communicable Disease Risk Factor Surveillance was adapted in Swahili. Therefore, fruit and vegetable intake aligned with the self-reported frequency of intake—described in days per week—rather than quantities or serving sizes to align with the WHO STEPS measurements. The survey collected demographic information, family history of NCDs, and lifestyle measurements, such as regular exercise, dietary patterns, smoking, and alcohol usage patterns. All participants were screened and documented for hypertension, obesity via measured body mass index (BMI), and HIV status [15]. Blood pressure (BP) was measured for all participants in a seated stance via the M4 Omron^®^ automatic BP machine to identify hypertension, as taken in the left arm three times. Hypertension was classified as systolic BP of ≥140 mmHg and diastolic BP of ≥90 mmHg [16].

Recommended daily intake of F&V was recommended to be 400 g (5–6 servings daily) [8]. This was based on the number of servings of F&V consumed per day in a typical week. Less than five servings a day was identified to be insufficient, and recommended fruit consumption was two or more servings. The recommended vegetable consumption was three or more servings per day. Central obesity was defined as waist circumference (WC) > 102 cm in men and >88 cm in women [17].

### 2.4. Measurements

Weight measurements were taken using a SECA^®^ weighing scale on a flat, hard surface. Participants were instructed to remove any heavy clothing (such as coats) and shoes, and to stand still on the weighing scale with their hands by their sides. The weighing scales were calibrated daily according to the manufacturer’s instructions.

Height was measured with a SECA^®^ stadiometer while the participant was facing directly ahead. Participants were instructed to remove their shoes, caps, or headscarves, keep their feet together, and stand with their arms by their sides. Measurements were taken with heels, buttocks, and upper back in contact with the stadiometer.

HIV diagnosis was conducted using immunochromatographic rapid tests: Determine HIV-1/2 (Alere Medical Co., Ltd., Tokyo, Japan) for screening, followed by UniGold HIV-1/2 (Trinity Biotech, Bray, Ireland) for confirmation of a positive result, as noted in the parent study in one hour [15]. Those noted as PWH in the community were referred to the BMC CTC for deeper investigation and ART medication referral.

### 2.5. Statistical Analysis

The original parenting data was preprocessed and summarized in the Stata statistical software version 15 (StataCorp, College Station, TX, USA). Numeric variables were characterized using their mean and standard deviation, while categorical variables were summarized using frequency and proportion. Demographic characteristics, as well as vegetable and fruit intake, were compared based on HIV status using Pearson’s Chi-squared test. To assess continuous variables based on HIV status, the Wilcoxon rank-sum test was employed.

Multinomial logistic regression was employed to determine factors influencing diverse fruit and vegetable intake levels. This resulted in two distinct binary logistic models. The first binary logistic model compares the intake of no to one day of fruits per week (fruits = (0–1)) to the intake of two to three days of fruits per week (fruits = (2–3)). The second binary logistic model compares not having one day of fruit intake per week (fruits = (0–1)) to the intake of four to seven days of fruits per week (fruits = (4–7)). The equations for the submodels are provided below:

$$Model\;1:\;log\left(\frac{fruits=\left(2–3\right)}{fruits=\left(0–1\right)}\right)=\sum_{j=0}^{k}\;\;\;\;\beta_{j}X_{j}$$

$$Model\;2:\;log\left(\frac{fruits=\left(4–7\right)}{fruits=\left(0–1\right)}\right)=\sum_{j=0}^{k}\;\;\;\;\beta_{j}X_{j}$$

Similarly, a multinomial logistic regression model was utilized to analyze vegetable intake. This yielded two binary logistic submodels. The first sub model contrasts the intake of no to one day of vegetables per week (vegetable = (0–1)) with the intake of two to three days of vegetables per week (vegetable = (2–3)). The second sub model compares no to one day of vegetable intake per week (vegetable = (0–1)) to the intake of four to seven days of vegetables (vegetable = (4–7)). The equations for these submodels are as indicated:

$$Model\;1:\;log\left(\frac{vegetable=\left(2–3\right)}{vegetable=\left(0–1\right)}\right)=\sum_{j=0}^{k}\;\;\;\;\beta_{j}X_{j}$$

$$Model\;2:\;log\left(\frac{vegetable=\left(4–7\right)}{vegetable=\left(0–1\right)}\right)=\sum_{j=0}^{k}\;\;\;\;\beta_{j}X_{j}$$

## 3. Results

### 3.1. Subject Characteristics by HIV Status

Table 1 provided an overview of respondent background characteristics based on their HIV status. Among those with Negative HIV status, 57% identified as Female and 43% as Male. In contrast, among individuals with Positive HIV status, 65% were Female, were 35% are Male. The relationship between level of education and HIV status was statistically significant (*p*-value = 0.012). Among Positive HIV cases, a majority (82%) had Primary or no formal education, while 13% had secondary education. Notably, there was no significant association between occupation and HIV status. For individuals with Negative HIV status, 64% were employed, and 36% were unemployed, while among Positive HIV cases, 69% were employed, and 31% were Uunemployed.

### 3.2. Fruit and Vegetable Intake by HIV Status

Table 2 revealed that the frequency of consuming fruits per week was notably associated with HIV status (*p*-value = 0.002). For individuals with Negative HIV status, 38% consumed fruits 0–1 days, 38% consumed them 2–3 days, and 25% consumed them 4–7 days, while for Positive HIV cases, 27%, 36%, and 38%, respectively, exhibited these patterns. However, no association was found between the frequency of eating vegetables and HIV status (*p*-value = 0.2). Among those with Negative HIV status, 9.7% consumed vegetables 0–1 days, 22% consumed vegetables 2–3 days, and 68% consumed vegetables 4–7 days. For Positive HIV cases, 6.2%, 19%, and 75%, respectively, followed these patterns.

WC:

- We used less than 102cm as low risk for men and above as high risk
- We used less than 88cm as low risk for women and above as high risk

Systolic BP was classified as measurements of ≥140. Average systolic BP, which was significantly higher in individuals with Negative HIV status (143 mmHg) compared to Positive status (138 mmHg) (*p*-value = 0.010). Random blood glucose levels differed significantly, with Negative HIV status individuals having lower levels (5.05 mmol/L) compared to those with Positive status (5.68 mmol/L) (*p*-value < 0.001). While BMI distribution did not show a significant difference between the two HIV statuses, WC in females was associated with HIV status, indicating that 61% of those with a Negative HIV status were at high risk. Contrastingly, 38% of the Positive cases showed a high risk (*p*-value < 0.001). However, WC in males did not exhibit a significant association with HIV status. Lastly, systolic BP indicated a substantial difference between the two HIV statuses, with more individuals with a Negative HIV status having systolic BP (51%) compared to those with a Positive status (40%) (*p*-value = 0.012). These variables were included as covariates in the multinomial models assessing fruit and vegetable intake.

### 3.3. Measuring Body Mass Index (BMI), Blood Pressure (BP), Waist Circumference (WC), and Blood Glucose by HIV Status

Table 3 captures obesity metrics of the study using BMI, BP, and WC. This was stratified according to HIV status, along with characterization of the average systolic and diastolic levels and blood glucose results. The BMI was categorized into normal, overweight, and underweight sections with sex divided into high risk and low risk.

### 3.4. Fruits

Among individuals who tested positive, males had elevated odds of consuming fruits 2–3 times per week compared to 0–1 times per week (OR = 4.97, 95% CI: 2.18, 11.34), and similarly, they displayed significantly increased odds of consuming fruits 4–7 times per week compared to 0–1 times per week (OR = 5.63, 95% CI: 2.48, 12.79). Employed individuals demonstrated significantly higher odds of consuming fruits 2–3 times per week versus 0–1 times per week (OR = 2.42, 95% CI: 1.12, 5.21), and they also had substantial increases in odds of consuming fruits 4–7 times per week compared to 0–1 times per week (OR = 2.49, 95% CI: 1.16, 5.35). Moreover, individuals classified as overweight displayed significantly elevated odds of consuming fruits 2–3 times per week compared to 0–1 times per week (OR = 2.57, 95% CI: 1.21, 5.49), and similarly, they exhibited significantly heightened odds of consuming fruits 4–7 times per week compared to 0–1 times per week (OR = 3.85, 95% CI: 1.82, 8.14).

Among PWH, being male (OR 5.63, 95% CI, 2.48–12.79), being employed (aOR = 2.49, 95% CI 1.16–5.35), and being overweight (OR = 3.85, 95% CI 1.82–8.14) were each independently associated with consuming fruits ≥ 4 days per week compared with ≤1 day. Among PWoH, overweight status (OR = 2.16, 95% CI 1.19–3.93) was also associated with greater fruit intake (Table 4).

### 3.5. Vegetables

Among those who tested positive, employed individuals had significantly higher odds of consuming vegetables 4–7 per week vs. 0.1 per week. Contrastingly, few variables appeared significant. Meanwhile, PWH employment was associated with higher odds of consuming vegetables ≥ 4 days per week (OR = 9.21, 95% CI: 1.17, 72.71) (Table 5).

## 4. Discussion

The purpose of this secondary analysis was to review whether F&V consumption varied across HIV status for this population of older adults in this Tanzanian region, based on data collected in the parent study from December 2018 to May 2019. Despite the data collection prior to the COVID-19 pandemic, reviewing the nutritional findings of Tanzania is essential due to chronic disease and cardiovascular risks among PWH and their implications for employment, socioeconomic standings, and HIV therapeutic measures [18]. These factors continued to be significant regardless of changes in food accessibility. The hypothesis of this research was that PWH would consume less F&V than PWoH due to comorbidities of HIV and malnutrition, along with socioeconomic disadvantages, food insecurity, and inaccessibility. However, the actual results proved there were no statistically significant factors in F&V intake when viewing HIV status. Additionally, there was a similar nutritional intake between PWH and PWoH examined. In the cross-examination study, there was a 15% increase in fruit consumption for PWH 4–7 times weekly than the consumption of the PWoH individuals, disagreeing with our initial presumption. These results point to the possibility that HIV status may not be a primary factor in F&V intake due to changes in HIV care and nutrient support in northwestern Tanzania or increased F&V accessibility to F&V despite socioeconomic status.

Multinomial regression analysis showed a positive association between higher BMI, male sex, and employment with increased fruit consumption (≥4 weekly) among PWH. While vegetable consumption did not differ strongly between PWH and PWoH groups, employment conveyed statistically significant associations with increased F&V intake in both groups. In the investigation of how fruit consumption was higher in PWH than PWoH for 4–7 times weekly, and the vegetable associations found, the findings emphasized how employment, and therefore financial security, can be a critical characteristic and hold more control compared to other socioeconomic factors, such as environment, access, and social inequality, when combating food insecurity in northwestern Tanzania.

Despite Tanzania’s nutritional improvement in the context of anemia and growth stunting, the increased urbanization of Tanzania in more rural locations, where HIV may have higher burdens, has increased nutritional diversity in one’s diet. In areas of the northwest, such as Shinyanga, case trials since the 2000s confirm how food and financial aid interventions under the supervision of the World Food Programme have supported nutritional assessment and counseling (NAC) among adults susceptible to food insecurity. There remains promise in sustainable interventions that are incorporated into daily HIV care [19]. Other under resourced environments indicate similar possibilities, endorsing the integration of nutritional diversity into HIV NAC in Tanzania [20]. If sustained, these measures can offer closure and more evidence as to why HIV status lacks intense associations with F&V differences in this cohort. Data from additional regions across the continent reinforce these findings, with the average F&V intake in Uganda at 1.4 daily servings, based on the 2014 STEPS Survey [21]. Likewise, in a cross-sectional study in Ethiopia, it was reported that nearly 75% of PWH adults consumed F&V less than once daily [22]. In viewing these patterns across various countries in Africa, it is uncommon for wider gaps between PWH and PWoH to occur, even with respect to socioeconomic status.

Other demographic and socioeconomic conditions still convey connections with F&V intake. As employment was positively related to fruit consumption, there is a consensus that financial stability and income increase one’s accessibility to nutrition [23]. A systematic meta-analysis published this year discovered that lower income, unemployment, and shorter ART periods were correlated with lower nutritional diversity [24]. Similarly, studies in multiple African settings have documented that diet quality among PWH is generally low, dominated by starchy staples and limited consumption of fruits and vegetables [25]. Fruit intake was reportedly higher in those with increased BMI, which may be motivated by personal weight management goals and other potential health conditions. Further, women of the PWoH category have higher BMI and central obesity, a previous observation with studies of body composition in East African diets [26]. Because of the risk of non-communicable disease among the aging demographic within sub-Saharan Africa, F&V accessibility for adults is essential. NAC and education-related interventions on HIV care have potency in improving nutritional intake outcomes and regulate BMI [20]. For instance, food-based vouchers and meal assistance plans can improve F&V intake for adults in both sectors of PWH and PWoH. A scoping review summarized how F&V consumption in Tanzania continues to fall short [27].

The parent study consisted of several limitations. Data on F&V intake were self-reported and, therefore, subjected to social desirability limitations upon recording, including overestimations or underestimations of food consumption due to memory within older populations or in attempts to align with perceived social norms. Moreover, limitations in the cross-sectional design imply causality between nutritional consumption and HIV status. Location-wise, the study was administered in one region local to Lake Victoria in Tanzania; therefore, results are only based in northwestern Tanzania and are not to be generalized to Tanzania entirely or any other African regions without considering discrepancies in economy, food systems, and agricultural context. Seasonality is another limitation of consideration, as PWH and PWoH recruitment was not conducted in parallel from December to May, where fruit consumption variances can be due to fruit availability during rainy and harvest seasons. Because this is a secondary analysis, omitted variables include health education and preventative counseling among PWoH recruited in the community. This may result in a confounding variable of differentiable healthcare engagement that could not be directly investigated in the parent study.

Upon important review of the discussed limitations, this work supports that HIV status is not the sole predictor for fruit or vegetable intake within older adults of northwestern Tanzania. Further studies are recommended for conducting multi-site designs or longitudinal investigations on nutritional consumption trends over longer durations for determining specific F&V produce most consumed per region, community-based interventions based on public policy—such as financial assistance programs—and prolonged food-based programs and their efficacy across aging citizens.

## 5. Conclusions

This study discovered that older PWH in Northwestern Tanzania indicated increased fruit consumption in comparison to older PWoH in Northwestern Tanzania. Central obesity was more common in women PWoH, delineating nutritional demands and programs for vulnerable populations with the PWH and PWoH cohorts of this study. These findings can prove practical to policymakers, health professionals, and research institutions to implement NAC and risk surveillance into HIV care and support programs, all while scaling obesity and weight management programs for PWoH.

## Figures and Tables

**Table 1:** Respondent background characteristics by human immunodeficiency virus (HIV) status.

Variable	HIV Status—Without HIV (0), With HIV (1)		*p*-Value ^2^
	0, *n* = 277 ^1^	1, *n* = 260 ^1^	
Sex			0.046
Female	158 (57%)	167 (65%)	
Male	119 (43%)	88 (35%)	
Level of education			0.012
College	6 (2.2%)	13 (5.0%)	
Primary or none	252 (91%)	214 (82%)	
Secondary	19 (6.9%)	33 (13%)	
Occupation			0.2
Employed	177 (64%)	180 (69%)	
Unemployed	100 (36%)	80 (31%)	

^1^ *n* (%) ^2^ Pearson’s Chi-squared test.

**Table 2:** Eating habits—fruit and vegetable (F&V) intake by HIV status.

Variable	HIV Status—Without HIV (0), With HIV (1)		*p*-Value ^2^
	0, *n* = 277 ^1^	1, *n* = 260 ^1^	
Number of days of fruits consumption in a week			0.002
0–1	104 (38%)	69 (27%)	
2–3	105 (38%)	92 (36%)	
4–7	68 (25%)	98 (38%)	
Number of days of vegetables consumption in a week			0.2
0–1	27 (9.7%)	16 (6.2%)	
2–3	61 (22%)	50 (19%)	
4–7	189 (68%)	194 (75%)	

^1^ *n* (%) ^2^ Pearson’s Chi-squared test.

**Table 3:** BMI, BP, WC, and blood glucose by HIV status.

Characteristic	HIV Status—Without HIV (0), With HIV (1)		*p*-Value ^2^
	0, *n* = 277 ^1^	1, *n* = 260 ^1^	
Average systolic (mmHg)	143 (26)	138 (26)	0.010
Average diastolic (mmHg)	85 (13)	87 (14)	0.11
Random blood glucose (mmol/L)	5.05 (1.42)	5.68 (1.17)	<0.001
BMI			0.095
Normal	138 (50%)	139 (53%)	
Overweight	122 (44%)	95 (37%)	
Underweight	17 (6.1%)	26 (10%)	
WC (Female)			<0.001
High risk	94 (61%)	62 (38%)	
Low risk	61 (39%)	103 (62%)	
WC (Male)			0.6
High risk	15 (13%)	9 (10%)	
Low risk	102 (87%)	79 (90%)	
Systolic BP			0.012
No	134 (49%)	155 (60%)	
Yes	141 (51%)	105 (40%)	

^1^ Mean (SD); *n* (%) ^2^ Wilcoxon rank sum test; Pearson’s Chi-squared test.

**Table 4:** Parameter estimates for fruit intake for people with and without HIV.

Characteristics	Negative		Positive	
	2–3/Week of Fruits Intake	4–7/Week of Fruits Intake	2–3/Week of Fruits Intake	4–7/Week of Fruits Intake
	aOR (95% CI)	aOR (95% CI)	aOR (95% CI)	aOR (95% CI)
Sex (male vs. female)	1.49 (0.83, 2.68)	1.87 (0.98, 3.59)	4.97 (2.18, 11.34)	5.63 (2.48, 12.79)
Employed vs. unemployed	0.86 (0.48, 1.52)	0.64 (0.33, 1.26)	2.42 (1.12, 5.21)	2.49 (1.16, 5.35)
Systolic BP (yes vs. no)	1.06 (0.60, 1.85)	1.10 (0.58, 2.07)	1.26 (0.64, 2.48)	0.97 (0.49, 1.91)
Overweight vs. normal	2.16 (1.19, 3.93)	1.75 (0.90, 3.41)	2.57 (1.21, 5.49)	3.85 (1.82, 8.14)
Underweight vs. normal	1.14 (0.38, 3.41)	0.43 (0.09, 2.17)	1.00 (0.30, 3.31)	1.45 (0.46, 4.59)

**Table 5:** Parameter estimates for vegetable intake for people with and without HIV.

	Negative		Positive	
Characteristics	2–3/Week of Vegetables Intake	4–7/Week of Vegetables Intake	2–3/Week of Vegetables Intake	4–7/Week of Vegetables Intake
	aOR (95% CI)	aOR (95% CI)	aOR (95% CI)	aOR (95% CI)
Sex (male vs. female)	1.37 (0.52, 3.60)	1.34 (0.57, 3.16)	3.45 (0.94, 12.59)	2.06 (0.62, 6.86)
Employed vs. unemployed	0.72 (0.27, 1.90)	0.95 (0.41, 2.21)	6.44 (0.75, 55.47)	9.21 (1.17, 72.71)
Systolic BP (yes vs. no)	2.25 (0.88, 5.78)	1.12 (0.49, 2.55)	1.51 (0.47, 4.88)	0.97 (0.33, 2.85)
Overweight vs. normal	1.23 (0.47, 3.26)	1.33 (0.56, 3.12)	1.57 (0.42, 5.88)	2.45 (0.73, 8.18)
Underweight vs. normal	2.15 (0.22, 21.27)	1.93 (0.23, 16.04)	1.07 (0.21, 15.29)	1.79 (0.21, 15.29)

## Data Availability

The authors confirm that the data supporting the findings of this study are available in the 2022 parent article. Raw data can be made available upon reasonable request from the corresponding author due to privacy.

## Abbreviations

The following abbreviations are used in this manuscript:

HIV	Human immunodeficiency virus
PWH	People with HIV
PWoH	People without HIV
F&V	Fruit and vegetable intake
CVD	Cardiovascular disease
WHO	World Health Organization
NCD	Non-communicable disease
LMIC	Low- and middle-income countries
AIDS	Acquired immunodeficiency syndrome
ART	Antiretroviral therapy
BMC	Bugando Medical Center
CTC	Care and Treatment Center
BMI	Body mass index
BP	Blood pressure
WC	Waist circumference
NAC	Nutritional assessment and counseling
